# Single immunization with an inactivated vaccine protects sheep from Schmallenberg virus infection

**DOI:** 10.1186/s13567-014-0079-6

**Published:** 2014-08-03

**Authors:** Silke Hechinger, Kerstin Wernike, Martin Beer

**Affiliations:** 1 Institute of Diagnostic Virology, Friedrich-Loeffler-Institut (FLI), Suedufer 10, Greifswald–Insel Riems, 17493, Germany

## Abstract

The arthropod-borne Schmallenberg virus (SBV), family *Orthobunyaviridae*, emerged in Europe in 2011. SBV is associated with a mild disease in adult ruminants but fetal malformation after an infection during a critical phase of pregnancy. A number of inactivated vaccines have been developed; their efficacy after two injections was demonstrated. To make the vaccination of sheep more efficient and economic the effect of a single immunization with one of these vaccines was investigated in the present study. Five vaccinated sheep and five additional control sheep were inoculated with SBV three weeks after vaccination and the results of a competitive ELISA, a standard microneutralization test and an SBV-specific real-time RT-PCR confirmed vaccine efficacy by demonstrating complete inhibition of viral replication in immunized animals.

## Introduction, methods and results

A previously unknown pathogen from the family *Orthobunyaviridae* emerged in Europe in autumn 2011 and was named Schmallenberg virus (SBV) according to the location of its discovery [1]. Midges (Culicoides spp.) are involved in its transmission [2]–[4]. While disease was first observed in cattle, sheep and goats, infection has also been detected in deer, bison, alpaca, moose and other wild ruminants [5]. The clinical picture is characterized by mild febrile disease in adult ruminants and the potential development of fetal malformations after transplacental infection [6]–[9]. An SBV-infection can be confirmed through detection of viral RNA both in serum during the first week post infection and in tissue samples [10].

As an effective instrument for disease control different inactivated vaccines have been developed and tested [11]. Besides, two commercial inactivated vaccines have already been granted a provisional marketing authorization in the United Kingdom and France, respectively [12],[13].

Until now, only studies about a protective effect after two vaccinations have been published. Reduction to a single injection minimizes workload and costs, which is especially important for sheep owners, as the animals are usually individually caught and restrained on the pastures for every injection. Therefore, the influence of a single immunization on a subsequent SBV-inoculation of sheep was investigated in the present study.

Five SBV-negative yearling sheep (S01 to S05) of European domestic breeds received a single subcutaneous injection with 2 mL of the MA-HT prototype vaccine from a previous study [11]. Five additional control sheep (S06 to S10) were left unvaccinated.

Three weeks after vaccination all animals were inoculated with 2 × 0.5 mL of calf serum containing an SBV field strain that was only passaged in the natural host. The production of this infectious serum has been described earlier [10]. The serological status was monitored weekly by a blocking ELISA (ID Screen® Schmallenberg virus Competition, ID vet, France) and a standard microneutralization test (SNT) [14]. Additionally, blood samples were taken daily on the 8 days following challenge infection and tested by ELISA and an SBV-specific reverse transcription real-time PCR (RT-qPCR) including an external standard based on the small (S) genome segment [15]. Rectal body temperatures were recorded daily during the entire study and the animals were examined daily for clinical signs. Autopsy was conducted three weeks after challenge infection and samples of spleen, mesenteric and mandibular lymph node and tonsils were taken and tested by RT-qPCR.

According to German legislation the experimental protocol was reviewed by a state ethics commission and has been approved by the competent authority (State Office for Agriculture, Food Safety and Fisheries of Mecklenburg-Vorpommern, Rostock, Germany. Ref. No. LALLF M-V TSD/7221.3-1.1-004/12).

The SBV-antibody-ELISA was used according to the manufacturer’s instructions. Results were calculated as the ratio of the optical density (OD) of the sample and the OD of the negative control (S/N, %). Samples with an S/N-value of 40% or less were considered positive. SNT-titers were calculated as reciprocal of the serum dilution showing 50% virus neutralization (neutralizing dose 50, ND50). Titers of 5 or more were considered positive. The MagAttract Virus Mini M48 Kit (Qiagen. Germany) was used to extract nucleic acid from serum and tissue samples according to the manufacturer’s recommendations.

All animals were serologically and virologically negative for SBV on the day of immunization. Vaccination was not associated with adverse side effects. None of the animals showed clinical signs of disease after challenge infection; the rectal body temperature remained within a normal range and autopsy did not reveal any gross pathological lesions.

On the day of challenge infection all five control animals were negative for SBV in both serological tests (Table 1). First SBV-specific antibodies were detected in controls on day 7 after challenge by SNT and on day 8 after challenge by ELISA. Neutralizing titers rose to values between 33 and 266 ND50 until day 21 post infection (Table 1).

**Table 1:** Serological results

**Animal**	**Group**	**SNT**^**a**^						**ELISA**^**a**^						**qRT-PCR**
		**0 dpv**	**14 dpv**	**0 dpc**	**7 dpc**	**14 dpc**	**21 dpc**	**0 dpv**	**14 dpv**	**0 dpc**	**7 dpc**	**14 dpc**	**21 dpc**	**tissue**
S01	vac	< 5	**17**	**12**	**10**	**10**	**7**	91.6	51.5	53.4	59.6	56.5	53.5	-
S02	vac	< 5	**56**	**24**	**14**	**10**	**14**	92.0	**45.8**	54.9	55.1	65.6	70.2	-
S03	vac	< 5	**12**	**7**	**6**	**7**	**14**	97.1	50.8	60.1	64.7	51.3	50.1	-
S04	vac	< 5	< 5	< 5	**7**	**7**	**7**	84.4	**33.0**	**42.4**	**46.0**	51.8	56.6	-
S05	vac	< 5	**20**	**28**	**14**	**12**	**12**	92.8	**47.5**	54.7	57.3	54.6	88.4	-
S06	co	ND	ND	< 5	**14**	**67**	**40**	ND	ND	92.5	**42.5**	**26.9**	**26.5**	+
S07	co	< 5	< 5	< 5	**12**	**375**	**266**	94.9	89.6	94.8	**43.8**	**14.6**	**17.5**	+
S08	co	< 5	< 5	< 5	**8**	**160**	**67**	95.9	86.7	92.0	**30.2**	**30.6**	**37.9**	+
S09	co	< 5	< 5	< 5	**10**	**224**	**160**	99.6	91.0	97.3	**44.5**	**23.1**	**21.9**	+
S10	co	< 5	< 5	< 5	**8**	**56**	**33**	82.4	67.9	73.9	58.8	**32.3**	**27.6**	+

Key serological results for vaccinated (vac) and control (co) animals are presented. Serological results are given for 0 and 14 days post vaccination (dpv) to 21 days post challenge infection (dpc). Neutralizing titers (SNT) are given as the reciprocal of the serum dilution showing 50% virus neutralization. Titers of 5 or more were considered positive. ELISA results are calculated as the ratio of the optical density (OD) of the sample and the OD of the negative control (S/N, %). Samples with an S/N value of 40% or less are considered positive. Results for RNA detection are given for tissue samples obtained at autopsy.

^a^Positive or doubtful ELISA results and positive SNT results are highlighted by bold figures.

All vaccinated animals were positive for SBV prior to challenge infection in at least one serological test (Table 1) and, thereafter, neutralizing titers remained largely constant with values between 7 and 14 ND50 on day 21 post challenge. The first antibodies were detected by SNT in S02 on day 7 after vaccination while the ELISA gave negative results for this animal for all sampling dates except day 14 after vaccination (Table 1). Samples of S01, S03 and S05 gave positive results in the SNT starting from day 14 after vaccination while S01 and S03 scored negative in the ELISA throughout the study. S05 gave only one doubtful ELISA result on day 14 post vaccination. In S04 neutralizing antibodies were detectable only one week after challenge infection but it scored positive in the ELISA on day 14 after vaccination and doubtful on the day of challenge infection.

After challenge infection, SBV-RNA was detectable in serum samples of all control animals for at least 3 consecutive days (Table 2). The mean maximum genome load in serum samples was 9.2 × 10^4^ genome copies per mL. Most tissue samples of the control animals gave positive PCR results as well. Only tonsils and mesenteric lymph nodes of S10, and mandibular lymph nodes of S06 scored negative. Mean genome loads per gram organ weight were 1.2 × 10^4^ copies/g for mandibular lymph nodes (minimum value: 4.9 × 10^2^ copies/g; maximum value: 3.6 × 10^4^ copies/g), 8.7 × 10^4^ copies/g for mesenteric lymph nodes (min: 5.9 × 10^1^; max: 3.4 × 10^5^), 1.2 × 10^5^ copies/g for spleens (min: 6.8 × 10^3^; max: 3.9 × 10^5^) and 1.6 × 10^5^ copies/g for tonsils (min: 4.7 × 10^1^; max: 6.0 × 10^5^).

**Table 2:** RNA detection in serum post challenge

**Animal**	**Group**	**Number of SBV-RNA copies per mL serum**								
		**0 dpc**	**1 dpc**	**2 dpc**	**3 dpc**	**4 dpc**	**5 dpc**	**6 dpc**	**7 dpc**	**8 dpc**
S 06	co	-	-	4.5 × 10^4^	3.9 × 10^3^	2.2 × 10^3^	ND	-	-	-
S 07	co	-	-	1.9 × 10^3^	1.2 × 10^4^	7.0 × 10^5^	ND	1.4 × 10^3^	-	-
S 08	co	-	-	1.4 × 10^4^	1.5 × 10^4^	2.5 × 10^4^	ND	-	-	-
S 09	co	-	-	5.2 × 10^2^	3.3 × 10^4^	1.5 × 10^5^	ND	-	-	-
S 10	co	-	-	8.5 × 10^2^	1.8 × 10^3^	6.4 × 10^3^	ND	-	-	-

Results are given for 0 to 8 days post challenge (dpc) for control animals (co). Dashes represent negative PCR results of the respective samples. Viral RNA was not detected in the serum of vaccinated animals at any time. Therefore, vaccinated animals were not included in the table. From 5 dpc serum samples were not available. Consequently, the RNA load could not be determined (ND) for this time point.

In contrast, viral RNA was not detected in any serum or tissue sample from the vaccinated animals.

## Discussion

In the unvaccinated control animals PCR results demonstrated viral replication and dissemination. The serological results support this observation as the SBV-infection induced a pronounced humoral immune response. In all vaccinated sheep, on the other hand, the absence of RNAemia demonstrates the protective effect of immunization. Furthermore, the antibody titers remained constant which suggests that the virus is eliminated before a memory immune response with an antibody boost could be triggered. The latter is in accordance with results of an earlier study in cattle [14] during which constant neutralizing titers were detected in seropositive animals after a second experimental SBV-infection. Interestingly, the single shot vaccination was highly efficacious and could even prevent both viremia and infection of target tissues such as mesenteric lymphnodes.

Interestingly, neutralizing titers are very low in vaccinated animals in this study and only a few ELISA results of their serum samples exceed the cut-off value. Thus, further factors, e.g. a cellular immune response, may contribute to the protective effect of vaccination. Similar observations have been reported for Rift Valley Fever virus (RVFV, family *Bunyaviridae*, genus Phlebovirus). Neutralizing antibodies are primarily responsible for protection against RVFV-infection [16]. However, one of six lambs treated with an inactivated vaccine showed a reduction in viremia and lack of clinical symptoms although detectable neutralizing antibodies were missing at the time of infection [17]. Furthermore, a study on Crimean-Congo hemorrhagic fever virus (CCHFV, family *Bunyaviridae*, genus Nairovirus) reports that an inactivated vaccine is able to elicit a considerable T-cell reaction in humans as measured by IFN-gamma production [18].

Unfortunately, there are no immunological studies available which deal with orthobunyavirus vaccines. Thus, the exact mechanism underlying our observations remains unclear. However, as saponins are able to stimulate cellular immune responses [19], this adjuvant used for the formulation of the vaccine may be an important factor for vaccine efficacy and protection from SBV-replication post challenge infection.

In conclusion, the present study demonstrated the complete protection of sheep from SBV-infection after a single injection while the underlying immunological mechanism needs to be further investigated.

## Competing interests

The authors declare that they have no competing interests.

## Authors’ contributions

Conceived and designed the experiments: KW, MB. Performed the experiments: SH, KW. Analyzed the data: SH, KW. Wrote the paper: SH, KW, MB. All authors read and approved the final manuscript.
